# Iron coordination in liquid FeAl_2_O_4_

**DOI:** 10.1098/rsta.2022.0351

**Published:** 2023-10-16

**Authors:** James W. E. Drewitt, Adrian C. Barnes, Sandro Jahn, Richard A. Brooker, Louis Hennet, Daniel R. Neuville, Henry E. Fischer

**Affiliations:** ^1^ School of Physics, University of Bristol, H H Wills Physics Laboratory, Tyndall Avenue, Bristol BS8 1TL, UK; ^2^ School of Earth Sciences, University of Bristol, Wills Memorial Building, Queens Road, Bristol BS8 1RJ, UK; ^3^ Institute of Geology and Mineralogy, University of Cologne, Zuelpicher Strasse 49b, Cologne 50674, Germany; ^4^ Interfaces, Confinement, Matériaux et Nanostructures (ICMN), CNRS, Université d’Orléans, Orléans Cedex 2 45071, France; ^5^ Conditions Extrêmes et Matériaux: Haute Température et Irradiation (CEMHTI), CNRS, Université d’Orléans,Orléans Cedex 2 45071, France; ^6^ Géomatériaux, Institut de Physique du Globe de Paris (IPGP), CNRS, Institut de physique du globe de Paris, Université de Paris Cité,1 rue Jussieu, Paris 75005, France; ^7^ Institut Laue-Langevin, 71 Avenue des Martyrs, CS 20156,Grenoble Cedex 9 38042, France

**Keywords:** high temperature, liquid structure, neutron diffraction, molecular dynamics, levitation, iron coordination

## Abstract

The structure of aerodynamically levitated liquid ${\text{FeAl}}_{2}{\text{O}}_{4}$ was measured by neutron diffraction with isotope substitution (NDIS). Classical and *ab initio* molecular dynamics simulations were performed and their results were found to be in close agreement with each other and the NDIS data. The results reveal that molten ${\text{FeAl}}_{2}{\text{O}}_{4}$ may be considered as an ionic liquid without any preference for particular short-range structural motifs.

This article is part of the theme issue ‘Exploring the length scales, timescales and chemistry of challenging materials (Part 1)’.

## 1. Introduction

As one of the most abundant elements on Earth, iron is a major constituent of natural magmas. The ability of iron to exist in different valence states can play a crucial role in altering the physical properties, such as viscosity, density and melting point, of the geological melts that control geochemical processes in the Earth’s deep interior [1–3]. Due to its important geological role, extensive experimental and simulation studies have been conducted to understand how iron is incorporated in the structure of oxide liquids and glasses (see reviews [4–8] and references therein). However, despite this extensive body of research, the local coordination of Fe by oxygen in these melts remains uncertain. Traditionally, particularly for silicate systems, comparisons are made with mineral structures. Strong fourfold (tetrahedral) or sixfold (octahedral) structural motifs are assumed for the liquid, with the former dominantly associated with ${\text{Fe}}^{3+}$ taking a similar role to fourfold coordinated ${\text{Al}}^{3+}$, and the latter mainly associated with ${\text{Fe}}^{2+}$. However, XANES studies on silicate melts suggest an average of fivefold coordination for a wide range of ${\text{Fe}}^{2+}/{\text{Fe}}^{3+}$ ratios [2,9–11], only moving towards fourfold at the highest ${\text{Fe}}^{3+}$ contents. The dominance of the fivefold average over a considerable range of ${\text{Fe}}^{2+}/{\text{Fe}}^{3+}$ ratios could result from all Fe being fivefold coordinated (as seen in some minerals) or a near constant mix of fourfold and sixfold coordination, with or without fivefold units [9]. However, although X-ray absorption and Mössbauer spectroscopy are the most widely employed techniques to determine iron coordination in geological glasses and melts [4], their reliance on indirect interpretation of spectra based on crystalline calibration data is a major limitation. Comparison between mineral analogues and melt structures is unreliable for compositions outside of the calibrated silicate range and risky even for simple iron oxides [12]. By contrast, diffraction techniques offer direct measurement of the local structure in liquids and glasses and are hence a valuable but underutilized tool in this field [12–14].

In this paper, we have employed the method of neutron diffraction with isotope substitution (NDIS) to measure directly the Fe–O coordination environment of aerodynamically levitated liquid ${\text{FeAl}}_{2}{\text{O}}_{4}$ at 2200 K. The NDIS data are analysed in combination with classical and *ab initio* molecular dynamics simulations to obtain an accurate atomic-scale model of the liquid structure.

## 2. Theory

The coherent scattering intensity of liquid ${\text{FeAl}}_{2}{\text{O}}_{4}$ measured by neutron diffraction is represented by the total structure factor

2.1 $$F(Q)=\sum_{\alpha }^{3}\sum_{\beta }^{3}c_{\alpha }c_{\beta }b_{\alpha }b_{\beta }[S_{\alpha \beta }(Q)-1],$$

where c and b denote the atomic fraction and coherent neutron scattering length of chemical element α or β, $S_{\alpha \beta }(Q)$ is a Faber & Ziman [15] partial structure factor, and Q is magnitude of the elastic scattering vector. In this study, two ${\text{FeAl}}_{2}{\text{O}}_{4}$ samples were prepared that were identical in every respect except for their isotopic composition, containing either ${\;}^{54}$Fe or ${\;}^{\mathrm{nat}}$Fe (i.e. Fe in its natural isotopic abundance). Due to the contrast in neutron scattering lengths ($b_{\mathrm{nat}}=0.945(2)$ fm cf. $b_{54}=0.42(1)$ fm [16]) all $S_{\alpha \beta }(Q)$ functions involving Fe receive different weightings leading to observably different total structure factors ${\;}^{\mathrm{nat}}F(Q)$ and ${\;}^{54}F(Q)$ measured for the two samples.

All correlations not involving Fe are identical in both measurements and can be eliminated in the difference function

2.2 $$\begin{array}{rl} \Delta F_{\mathrm{Fe}}(Q) & \;\equiv {}^{\mathrm{nat}}F(Q)-{}^{54}F(Q)\\ & \;=2c_{\mathrm{Fe}}(b_{\mathrm{nat}}-b_{54})(c_{O}b_{O}[S_{\mathrm{FeO}}(Q)-1]+c_{\mathrm{Al}}b_{\mathrm{Al}}[S_{\mathrm{FeAl}}(Q)-1])\\ & \;\;+c_{\mathrm{Fe}}^{2}(b_{\mathrm{nat}}^{2}-b_{54}^{2})[S_{\mathrm{FeFe}}(Q)-1]. \end{array}$$

Similarly, all the correlations involving Fe and the chemical element μ≠Fe are eliminated in the difference function

2.3 $$\begin{array}{rl} \Delta F_{\mu }(Q) & \;\equiv \frac{b_{\mathrm{nat}}{}^{54}F(Q)-b_{54}{}^{\mathrm{nat}}F(Q)}{b_{\mathrm{nat}}-b_{54}}\\ & \;=2c_{\mathrm{Al}}c_{O}b_{\mathrm{Al}}b_{O}[S_{\mathrm{AlO}}(Q)-1]+c_{\mathrm{Al}}^{2}b_{\mathrm{Al}}^{2}[S_{\mathrm{AlAl}}(Q)-1]\\ & \;\;+c_{O}^{2}b_{O}^{2}[S_{\mathrm{OO}}(Q)-1]-c_{\mathrm{Fe}}^{2}b_{\mathrm{nat}}b_{54}[S_{\mathrm{FeFe}}(Q)]. \end{array}$$

The total pair distribution function G(r) provides a measure of the variation in atom–atom pair density as a function of distance r in real-space, and is determined by the Fourier transformation

2.4 $$\begin{array}{rl} G(r) & \;=\frac{1}{2\pi^{2}r\rho }\int_{0}^{\infty }Q[F(Q)]\sin(Qr)\;\text{d}Q\\ & \;=\sum_{\alpha =1}^{n}\sum_{\beta =1}^{n}c_{\alpha }c_{\beta }b_{\alpha }b_{\beta }[g_{\alpha \beta }(r)-1], \end{array}$$

where $g_{\alpha \beta }(r)$ is a partial pair distribution function. The mean coordination number, ${\bar{n}}_{\alpha }^{\beta }$, gives the average number of β atoms in a spherical coordination shell of radius $r_{1}\leq r\leq r_{2}$ centred on an atom of type α and is obtained by integrating over a peak in real-space arising from a specific $g_{\alpha \beta }(r)$ function, according to

2.5 $${\bar{n}}_{\alpha }^{\beta }=4\pi \rho c_{\beta }\int_{r_{1}}^{r_{2}}g_{\alpha \beta }(r)r^{2}\;\text{d}r.$$

It is important to recognize that the coordination number is not precisely defined. In general, peaks in G(r) overlap so that the contribution from neighbouring peaks cannot be unambiguously separated. Similarly, the coordination number is a statistical quantity that is an average so that it does not need to be an integer. Hence, we do not need to and it is not helpful to discuss atomic coordination in liquids in terms of strict and clearly coordinated structural motifs (tetrahedra, octahedra, etc.).

The real-space difference functions, denoted $\Delta G_{\mathrm{Fe}}(r)$ and $\Delta G_{\mu }(r)$, are obtained by Fourier transforming the corresponding reciprocal-space functions in equations (2.2) and (2.3), respectively, and are defined by replacing the $S_{\alpha \beta }(Q)$ functions in $\Delta F_{\mathrm{Fe}}(Q)$ and $\Delta F_{\mu }(Q)$ with their corresponding real-space partials $g_{\alpha \beta }(r)$.

## 3. Methods

### (a) Sample preparation

Polycrystalline FeO was synthesized by oxidizing pure Fe (Alfa Aesar, powder, spherical, <10 µm, 99.9%) or ${\;}^{54}$Fe (Pennywood chemicals, foil, 99.91% enrichment) in a gas-mixing vertical tube furnace at the University of Bristol petrology laboratory, using a $\text{CO}/{\text{CO}}_{2}$ mixture to control the oxygen fugacity ($fO_{2}$). The samples were heated in ${\text{Al}}_{2}{\text{O}}_{3}$ crucibles at 1173 K for approximately 48 h at an $fO_{2}$ of $10^{-14}$ bar. The samples were re-ground in an agate pestle and mortar periodically every 12 h during heat treatment to ensure sample homogeneity. The unit cell parameter for the synthesized ${\;}^{\mathrm{nat}}$FeO sample was measured using a Bruker D8 X-ray powder diffractometer giving a=4.2892 Å, corresponding to ${\text{Fe}}_{0.961}O$ stoichiometry [17].

The ${\;}^{\mathrm{nat}}{\text{FeAl}}_{2}{\text{O}}_{4}$ and ${\;}^{54}{\text{FeAl}}_{2}{\text{O}}_{4}$ samples were prepared by mixing the synthesized FeO or ${\;}^{54}\mathrm{FeO}$ with dried ${\text{Al}}_{2}{\text{O}}_{3}$ (Alfa Aesar, 99.995%) powder in the appropriate proportions in an agate pestle and mortar to achieve a powder grain size of ≲30 µm. The powder mixtures were heated for 2 h in Pt capsules at 1773 K at an $fO_{2}$ of $10^{-9.5}$ bar. To ensure there was no Fe loss to the Pt capsules, they were pre-saturated using Fe (Alfa Aesar, 99.9%) or ${\;}^{54}$Fe (AERE, Harwell) powders at 1273 K at an $f_{O_{2}}$ of $10^{-7.6}$ bar to form a Fe–Pt alloy skin on the interior of the Pt capsule [18]. Polycrystalline samples appeared dark green, as expected for Hercynite (${\text{Fe}}^{2+}{\text{Al}}_{2}{\text{O}}_{4}$). A mass of approximately 400 mg per sample was recovered cleanly from the Pt capsule. In preparation for the NDIS experiment, spherical samples of diameter ≲3 mm were pre-formed using aerodynamic levitation and laser-heating under a pure Ar gas flow at the CEMHTI laboratory in Orléans, France.

### (b) Neutron diffraction experiment

The neutron diffraction measurements were performed using the D4c neutron diffractometer [19] at the Institut Laue-Langevin research reactor in Grenoble, France. The experiment [20] was performed using a monochromatic beam of neutrons of wavelength 0.4961(1) Å and the aerodynamic levitation and ${\text{CO}}_{2}$ laser-heating apparatus developed by Hennet *et al.* [21]. To reduce background scattering from the sample environment, the 9 mm horizontal and 48 mm vertical incident beam was collimated vertically using two neutron absorbing ${\;}^{10}{\text{B}}_{4}\text{C}$ panels with 4 mm separation, positioned close to the sample position. The upper section of the conical levitation nozzle was machined from vanadium which has an almost entirely incoherent neutron scattering cross-section [16]. Prior to the liquid diffraction measurements, the instrument sample chamber was evacuated to $≲10^{-4}\;\text{mbar}$ and filled to atmospheric pressure with the levitation gas (Ar + 5 ppm ${\text{O}}_{2}$) to prevent oxidation of the sample or reduction to metallic Fe and to ensure a reproducible atmosphere for reliable background correction. The ≲3 mm diameter levitated samples were melted using two 125 W ${\text{CO}}_{2}$ lasers (Synrad 60-1) of wavelength 10.6 μm incident from above the sample at $20^{\circ }$ to the vertical axis and heated to a constant temperature of 2200(30) K, as measured by an optical pyrometer placed within the instrument sample chamber operating at a wavelength of 0.85 µm. Heating from two angles, combined with the continuous rotation of the levitated sample, reduced the temperature inhomogeneity due to cooling from the levitation gas to approximately 50 K at 2200 K on the lower portion of the sample, which is obstructed from the incident neutron beam by the ${\;}^{10}{\text{B}}_{4}\text{C}$ collimator [21]. During the measurements, the measured temperatures remained very stable within ±20 K. Neutron diffraction patterns of the levitated ${\;}^{\text{nat}}{\text{FeAl}}_{2}{\text{O}}_{4}$ and ${\;}^{\text{54}}{\text{FeAl}}_{2}{\text{O}}_{4}$ liquids were recorded for 5 h, with the full detector range scanned at intervals of 15–30 min. Crystallization events occasionally occurred in both samples upon which the experiments were paused to re-melt the sample before continuing the measurements. For each set of measurements, successive diffraction patterns for every full detector scan were compared and any measurements found to be inconsistent within the statistical noise were rejected. Two background diffraction measurements of the empty levitation apparatus were made, one inside the Argon-filled sample chamber with a continuous flow of levitation gas and one in the evacuated chamber. An additional measurement of a 5 mm diameter solid vanadium sphere, placed on top of the levitation nozzle within the evacuated diffraction chamber with a volume above the nozzle similar to the levitated sample volume illuminated by neutrons, was conducted for the purpose of normalization [22].

Before corrections the background scattering data from the levitation nozzle under vacuum were subtracted from the sample and the nozzle (with the levitation gas present) data. Corrections for the levitation gas (‘empty container’), self attenuation and Plazcek corrections for the samples and vanadium were applied [22]. Due to the small size of the sample, the attenuation corrections are small (less than 5% with little angular dependence) and any multiple scattering is negligible. A final, small, normalization correction (due to uncertainties in the scattering volume of the sample and vanadium sphere) was made to the data to ensure consistency with the known scattering lengths of the sample.

In addition to the nuclear scattering from the atomic nuclei there is also a contribution from the paramagnetic scattering of the electrons on the ${\text{Fe}}^{2+}$ ions that manifests itself as a Q dependent scattering contribution in the data. This contribution is unchanged in the two isotopically different samples and cancels in the first order difference. However, for the total structure factors and $\Delta F_{\mu }(Q)$ it appears as an unwanted slope in the data that gives unphysical contributions in G(r) at small r. For the case of ${\text{Fe}}^{2+}$ the orbital angular momentum (L) contribution to this scattering is small and we have taken the total angular momentum as J∼S [23] so that the magnetic cross-section may be taken as [24]

3.1 $$\frac{d\sigma }{d\Omega }≃4c_{M}{\left(\gamma r_{e}\right)}^{2}<j_{0}{>}^{2}(Q),$$

where

3.2 $$\begin{array}{rl} \langle j_{0}(Q)\rangle^{2} & \;=0.0263\exp(-34.9597Q^{2})+0.3668\exp(-15.9435Q^{2})\\ & \;\;+0.6188\exp(-5.5935Q^{2})-0.0119, \end{array}$$

where the coefficients have been taken from the International Table for Crystallography [25]. All analysis of the scattering data was made after this contribution had been removed.

### (c) Classical molecular dynamics

Molecular dynamics (MD) simulations of liquid ${\text{FeAl}}_{2}{\text{O}}_{4}$ were carried out using DLPOLY (v.3.17) [26]. Morse potentials for ${\text{Fe}}^{2+}$, ${\text{Al}}^{3+}$ and ${\text{O}}^{2-}$ ions were taken from the paper of Pedone *et al.* [27]. The simulations were carried out with 9996 atoms (1428 Fe, 2856 Al and 5712 O) giving the same stoichiometry as the samples. The simulation cubic box size was of length 49.83 Å corresponding to an atomic number density of 0.0808 Å^-3^. The simulation was run at 2500 K using a Berendsen *NVT* thermostat using 1 fs steps and was run for a total of 50 000 steps (50 ps) with 500 equilibration steps (0.5 ps) at the start.

The final MD configuration was used as the input configuration for a Reverse Monte Carlo (RMCPROFILE [28]) refinement of the structure to the measured diffraction data. The total structure factors ${\;}^{\text{nat}}F(Q)$ and ${\;}^{54}F(Q)$ measured for the two isotopic samples and the difference functions $\Delta F_{\mathrm{Fe}}(Q)$ and $\Delta F_{\mu }(Q)$ were included in the refinement. The refinement was run with approximately 350 000 moves generated after which the structure factors for the refined configuration had converged with the experimental data. No significant differences were observed between the MD and RMC refined data, demonstrating strong consistency between the classical MD model and the neutron diffraction measurements.

### (d) *Ab initio* molecular dynamics

*Ab initio* molecular dynamics (AIMD) simulations were performed using the Quickstep module of the CP2K code [29]. The particle interactions were treated in the frame of density functional theory with a mixed Gaussian and planewave approach [30]. A multi-grid with five levels was employed for real-space integration with a planewave cut-off of 800 Ry for the finest level and a relative cut-off for Gaussian mapping of 70 Ry. As basis sets, we chose the MOLOPT split valence double-zeta valence plus polarization parameters [31] supplied by CP2K. For computational efficiency, we employed Goedecker–Teter–Hutter pseudopotentials [32]. As the exchange-correlation functional, the generalized gradient approximation (GGA) according to Perdew *et al.* (PBE) [33] was used. Due to unpaired spins in the d-orbitals of Fe, the electronic structure calculations were done in spin-polarized mode. We performed simulations without and with Hubbard-U correction [34], the latter to account for the strong correlations between Fe d-electrons. While the resulting structure model from standard GGA was already in good agreement with the experiments, including +U did have a minor effect on the Fe–O and Fe–Fe correlations and a slight improvement in the overall agreement with the NDIS data. Here, we chose the same U−J parameter of 2.5 eV as in a previous study of Fe-bearing oxide melt [35]. In the following, only results of the GGA + U simulations are presented. The periodic simulation box contained 112 atoms, i.e. 16 formula units of ${\text{FeAl}}_{2}{\text{O}}_{4}$. Initial structures were adopted from earlier AIMD simulations of ${\text{CaAl}}_{2}{\text{O}}_{4}$ melts [36]. The cubic box length was set to 11.15 Å, and simulations were performed in the canonical ensemble at constant volume and temperature with a particle density of 0.0808 Å^-3^. The temperature of 2200 K was controlled by the canonical sampling through velocity rescaling method [37] using a time constant of 0.1 ps. The AIMD time step was set to 1 fs. After initial equilibration, structural properties were derived from a 20 ps production run.

## 4. Results

**Figure 1.**
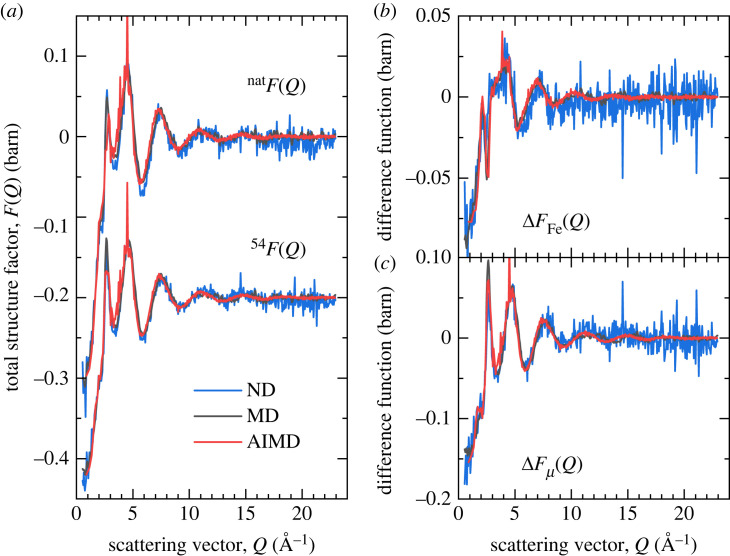
(*a*) Total structure factors ${\;}^{\mathrm{nat}}F(Q)$ and ${\;}^{54}F(Q)$ and difference functions (*b*) $\Delta F_{\mathrm{Fe}}(Q)$ and (*c*) $\Delta F_{\mu }(Q)$ of aerodynamically levitated liquid FeAl_2_O_4_ at 2200 K as measured using the D4C neutron diffractometer (blue) shown together with the corresponding functions computed from the MD (black) or AIMD (red) simulation trajectories. (Online version in colour.)

**Figure 2.**
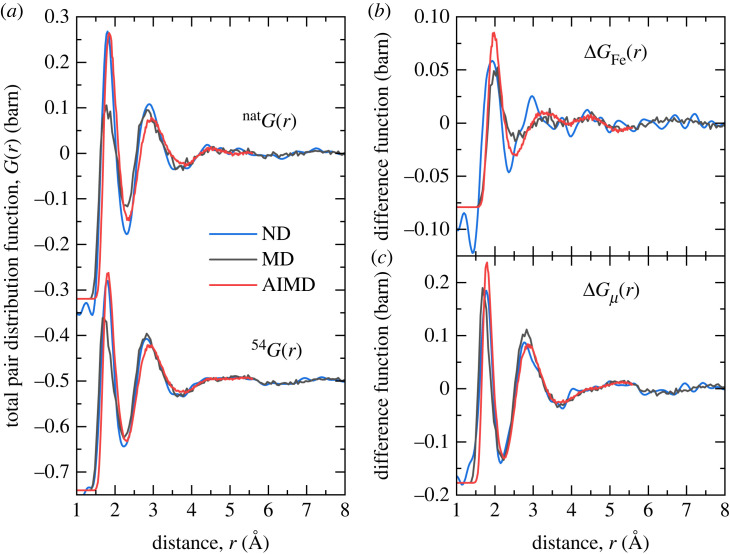
(*a*) Total pair distribution functions ${\;}^{\mathrm{nat}}G(r)$ and ${\;}^{54}G(r)$ and difference functions (*b*) $\Delta G_{\mathrm{Fe}}(r)$ and (*c*) $\Delta G_{\mu }(r)$ for aerodynamically levitated liquid FeAl_2_O_4_ at 2200 K as determined by Fourier transforming the corresponding reciprocal-space functions in figure 1 (blue), shown together with the corresponding functions computed from the MD (black) or AIMD (red) simulation trajectories. (Online version in colour.)

The total structure factors ${\;}^{\text{nat}}F(Q)$ and ${\;}^{54}F(Q)$ of the levitated ${\text{FeAl}}_{2}{\text{O}}_{4}$ liquids at 2200(30) K are shown in figure 1*a* together with the structure factors computed from the MD and AIMD simulation trajectories. Small discrepancies between the experimental and simulation results are observed in the region of the principal peak in F(Q), which relates to structural ordering on extended length scales [38]. These longer range correlations are not fully captured in the AIMD simulations due to the relatively small periodic simulation box of 112 atoms. A marked improvement in capturing these low-Q features is observed for the larger-scale classical MD simulations. The reciprocal-space difference functions $\Delta F_{\mathrm{Fe}}(Q)$ (equation 2.2) and $\Delta F_{\mu }(Q)$ (equation 2.3) are shown in figure 1*b*,*c*, respectively. The corresponding real-space functions ${\;}^{\text{nat}}G(r)$, ${\;}^{54}G(r)$, $\Delta G_{\mathrm{Fe}}(r)$ and $\Delta G_{\mu }(r)$ are shown in figure 2. Peak bond distances $r_{\mathrm{AlO}}$ (Å), $r_{\mathrm{FeO}}$ (Å), $r_{\mathrm{OO}}$ (Å) and average coordination numbers ${\bar{n}}_{\mathrm{Al}}^{O}$, ${\bar{n}}_{\mathrm{Fe}}^{O}$ obtained from the experimental and simulated real-space functions are reported in table 1. The NDIS, MD and AIMD results are in good overall agreement within the bounds of uncertainty, although the bond distances $r_{\mathrm{AlO}}$ and $r_{\mathrm{FeO}}$ determined are slightly more consistent with the experimental difference functions for the AIMD calculations than the classical MD results. In $\Delta G_{\mu }(r)$, all correlations involving Fe and the chemical element μ≠Fe are eliminated such that the first peak at 1.78(1) Å arises solely from nearest neighbour Al–O correlations. In $\Delta G_{\mathrm{Fe}}(r)$, there is no indication of any residual contribution from Al–O correlations, indicating all correlations not involving Fe are successfully eliminated. This provides a direct measurement of the nearest neighbour Fe–O peak at 1.93(3) Å which cannot be resolved in ${\;}^{\text{nat}}G(r)$ or ${\;}^{54}G(r)$. Although the $g_{\mathrm{AlO}}(r)$ and $g_{\mathrm{FeO}}(r)$ are successfully separated in the $\Delta G_{\mu }(r)$ and $\Delta G_{\mathrm{Fe}}(r)$ difference functions, they are not entirely isolated from other correlations, especially towards higher-r where contributions from other atomic pair interactions become more significant. Hence, the first minimum in $\Delta G_{\mu }(r)$ and $\Delta G_{\mathrm{Fe}}(r)$ does not correspond to the first minimum in $g_{\mathrm{AlO}}(r)$ and $g_{\mathrm{FeO}}(r)$, respectively, resulting in an underestimate of the calculated average coordination numbers due to the limited integration range.

**Table 1.** Real-space peak positions $r_{\mathrm{AlO}}$, $r_{\mathrm{FeO}}$, $r_{\mathrm{FeO}}$ from the NDIS ${\;}^{\text{nat}}G(r)$, ${\;}^{54}G(r)$ and difference function $\Delta G_{\mathrm{Fe}}(r)$, $\Delta G_{\mu }(r)$ measurements, and average coordination numbers ${\bar{n}}_{\mathrm{Al}}^{O}$, ${\bar{n}}_{\mathrm{Fe}}^{O}$ calculated using equation (2.5) up to the first minimum $r_{2}$ after the peak of interest. The corresponding values from the classical MD and AIMD simulation results and partial pair distribution functions $g_{\mathrm{AlO}}(r)$, $g_{\mathrm{FeO}}(r)$, $g_{\mathrm{OO}}(r)$ are also provided.

function	technique	$r_{\mathrm{AlO}}\;(\text{Å})$	$r_{\mathrm{FeO}}\;(\text{Å})$	$r_{\mathrm{OO}}\;(\text{Å})$	${\bar{n}}_{\mathrm{Al}}^{O}$	${\bar{n}}_{\mathrm{Fe}}^{O}$	$r_{2}$ (Å)
${\;}^{\text{nat}}G(r)$	NDIS	1.80(2)		2.89(5)			
${\;}^{\text{nat}}G(r)$	MD	1.77(2)		2.84(5)			
${\;}^{\text{nat}}G(r)$	AIMD	1.85(2)		2.95(5)			
${\;}^{54}G(r)$	NDIS	1.79(2)		2.81(5)			
${\;}^{54}G(r)$	MD	1.72(2)		2.83(5)			
${\;}^{54}G(r)$	AIMD	1.81(2)		2.90(5)			
$\Delta G_{\mu }(r)$	NDIS	1.78(1)		2.77(5)	4.34(5)		2.15(1)
$\Delta G_{\mu }(r)$	MD	1.70(1)		2.82(5)	3.83(5)		2.20(2)
$\Delta G_{\mu }(r)$	AIMD	1.79(1)		2.86(5)	4.16(5)		2.23(1)
$\Delta G_{\mathrm{Fe}}(r)$	NDIS		1.93(3)			3.26(5)	2.36(1)
$\Delta G_{\mathrm{Fe}}(r)$	MD		2.00(3)			3.40(5)	2.42(5)
$\Delta G_{\mathrm{Fe}}(r)$	AIMD		1.98(3)			3.78(5)	2.45(5)
$g_{\mathrm{AlO}}(r)$	MD	1.70(1)			4.25(5)		2.50(1)
$g_{\mathrm{AlO}}(r)$	AIMD	1.79(1)			4.40(5)		2.50(1)
$g_{\mathrm{FeO}}(r)$	MD		2.01(3)			4.95(5)	2.90(1)
$g_{\mathrm{FeO}}(r)$	AIMD		1.98(3)			4.94(5)	2.90(1)
$g_{\mathrm{OO}}(r)$	MD			2.78(3)			
$g_{\mathrm{OO}}(r)$	AIMD			2.78(3)			

**Figure 3.**
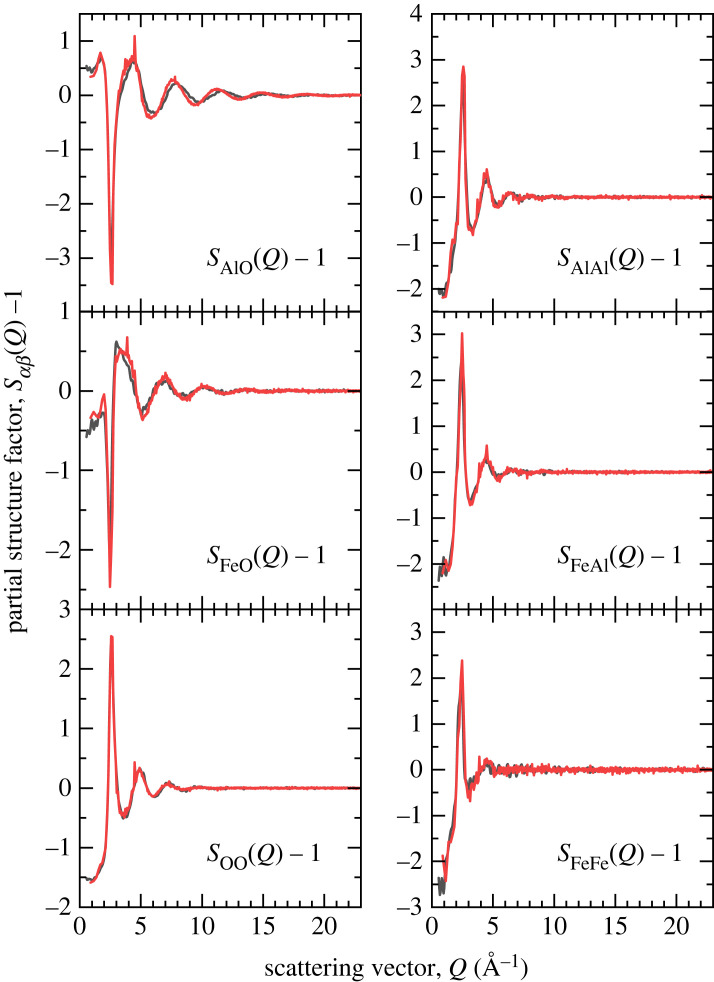
Faber–Ziman partial structure factors $S_{\alpha \beta }(Q)$ for liquid FeAl_2_O_4_ at 2200 K computed from the MD (black) or AIMD (red) simulation trajectories. (Online version in colour.)

**Figure 4.**
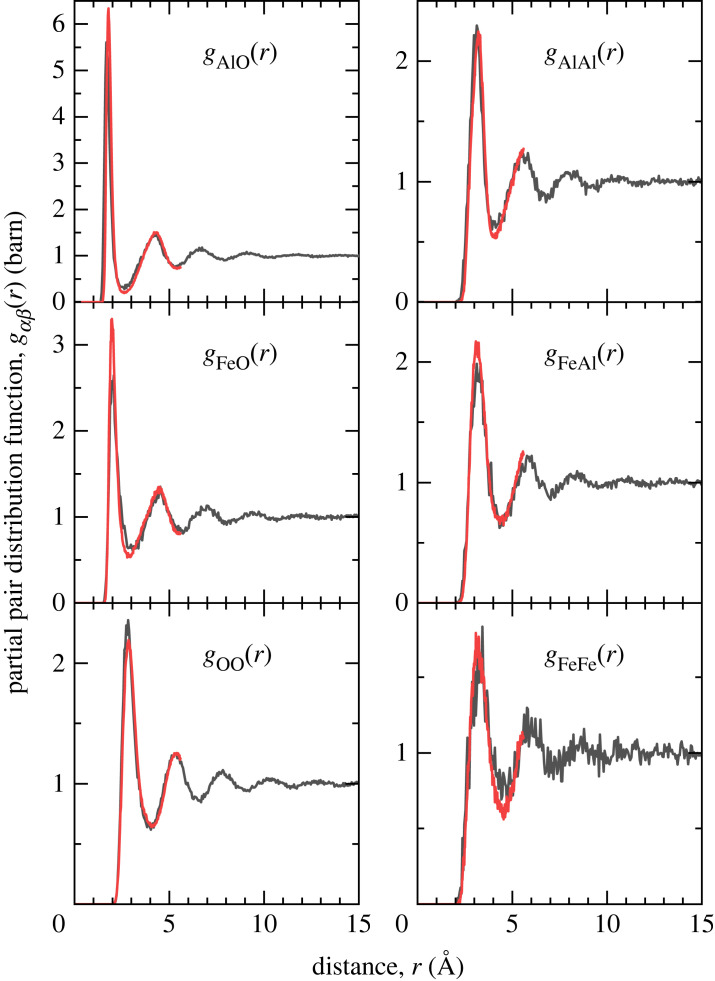
Partial pair distribution functions $g_{\alpha \beta }(r)$ for liquid FeAl_2_O_4_ at 2200 K computed from the MD (black) or AIMD (red) simulation trajectories. (Online version in colour.)

The Faber–Ziman partial structure factors $S_{\alpha \beta }(Q)$ and pair distribution functions $g_{\alpha \beta }(r)$ calculated from the atomic configurations generated by the MD and AIMD simulations are shown in figures 3 and 4, respectively. Considering the AIMD results, the first peak in $g_{\text{AlO}}(r)$ appears at 1.79(1) Å and gives an average coordination number ${\bar{n}}_{\mathrm{Al}}^{O}=4.40(5)$ when integrated to the first minimum at 2.50(1) Å. The first peak in $g_{\text{FeO}}(r)$ appears at 1.98(3) Å and gives an average coordination number of ${\bar{n}}_{\mathrm{Fe}}^{O}=4.94(5)$ when integrated to the first minimum at 2.90(1) Å.

## 5. Discussion

The results from the MD and AIMD simulations show strong similarities and good broad agreement with the NDIS data at both the total and difference function level (figures 1 and 2). The first peak in $g_{\text{AlO}}(r)$ at 1.79(1) Å is close to that observed in other aluminate oxide liquids and glasses [39–42]. However, there is a notable asymmetry of the peak on the high r side and the first minimum in $g_{\text{AlO}}(r)$ does not reach zero. A close analysis of the oxygen coordination around aluminium from the MD and AIMD simulations shows that, unlike most aluminate glasses in which aluminium is almost entirely fourfold coordinated by oxygen [40,41,43], there is a large variation in the coordination number around the individual aluminium atoms. Although the results show the predominance (55%) of fourfold coordinated species in the first coordination shell (defined by the first minimum in real-space), it is evident, unlike the case for aluminate glasses [40], that the Al–O structure cannot be considered as an extended network of corner shared ${\text{AlO}}_{4}$ tetrahedra but rather a dynamically varying local coordination consistent with a high temperature liquid [44].

**Figure 5.**
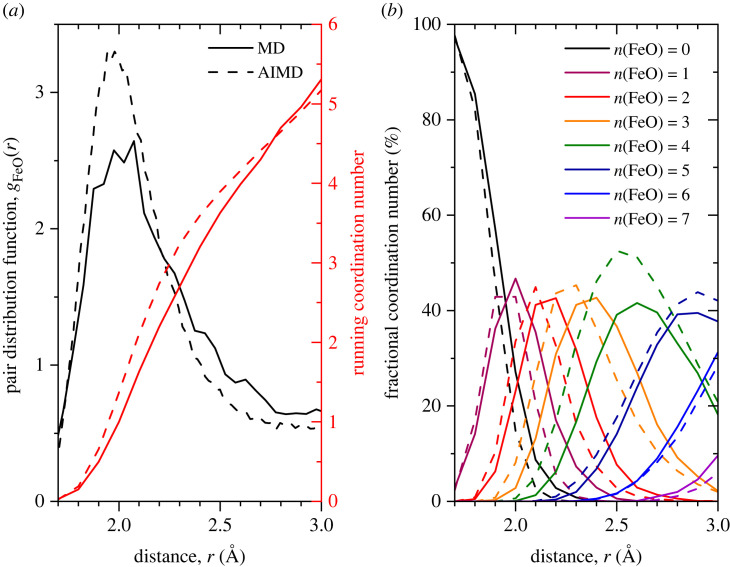
(*a*) Pair distribution function $g_{\mathrm{FeO}}(r)$ (black) and running coordination number (red) as calculated from the MD (solid curves) and AIMD (dashed curves) simulations. (*b*) Fraction of partial coordination numbers n(FeO) from 0 to 7 as a function of distance. (Online version in colour.)

The first peak observed in $g_{\text{FeO}}(r)$ at 1.98(3) Å, is consistent with the Fe–O distance associated with ${\text{Fe}}^{2+}$ ions [45]. It is clear from the far from zero value of $g_{\text{FeO}}(r)$ at the first minimum that well defined species of fixed coordination do not exist in the liquid. A close analysis of the oxygen coordination of the iron atoms in the MD and AIMD simulations (figure 5) shows that, even within the first coordination shell there is no coordination preference for oxygen atoms around iron atoms with large numbers of fourfold, fivefold and sixfold coordinated atoms present simultaneously. In the literature, the oxygen coordination around iron is often cited as the origin of many of the properties (viscosity, etc.) of iron-containing liquids. In particular, reference is frequently made to strong fourfold (tetrahedral) or sixfold (octahedral) structural motifs being present in the liquid [5]. In this study, we find no evidence for a dominant and fixed coordination but rather a continuous distribution of local environments. If strong bonding in rigid structural motifs was present we would expect considerable oscillatory structure out to high Q in $\Delta F_{\text{Fe}}(Q)$. This is clearly not observed. The results presented here are consistent with the conclusions of Alderman *et al.* [14], who similarly found a wide variation in the iron–oxygen coordination in liquid ${\text{Fe}}_{2}{\text{SiO}}_{4}$.

The close similarity between the MD and AIMD simulations and their good agreement with the experimental data supports a conclusion that liquid ${\text{FeAl}}_{2}{\text{O}}_{4}$ may be treated fundamentally as an ionic liquid. The potentials used in the MD simulation are ionic in nature and the simulation consisting of approximately 10 000 atoms in a box size of approximately 50 Å in length is sufficient to obtain a good representation of the medium range ordering of the ions. By contrast, the AIMD simulation, while containing considerably fewer atoms, gives more direct information about the short-range order (bonding) and in particular, it is possible to probe the electron distributions around the atoms as well. An examination of the electron distributions obtained from the AIMD simulation confirms that almost complete charge transfer has taken place between the Fe and Al atoms and the oxygen atoms with a small indication of some polarization of the electron distribution on the ${\text{O}}^{2-}$ ions. It is this almost complete charge transfer that explains the excellent agreement between the MD and AIMD results.

In summary, the close agreement between the experimental data and two independent simulations supports the conclusion that both the AIMD and MD methods give reliable atomistic models of the structure of liquid ${\text{FeAl}}_{2}{\text{O}}_{4}$. Furthermore, it supports the conclusion that this and other aluminate liquids may be considered as fundamentally ionic in nature with no requirement for strong, covalently bonded, structural motifs (i.e. tetrahedra or octahedra) to be present. Indeed, the results show little preference at all for a preferred local structure around the iron ions.

## 6. Conclusion

In this work, we have demonstrated, through close comparison of computer simulation and detailed structural measurements from neutron diffraction and isotopic substitution, that liquid ${\text{FeAl}}_{2}{\text{O}}_{4}$ may be considered as an ionic liquid consisting of essentially ${\text{Fe}}^{2+}$, ${\text{Al}}^{3+}$ and ${\text{O}}^{2-}$ ions. Unlike the case for aluminate glasses [40,41,43], there is no extended network of corner shared ${\text{AlO}}_{4}$ tetrahedra but rather a dynamically varying local coordination. By contrast to traditional interpretations of the coordination environment of iron in silicate melts, the Fe in this liquid cannot be considered in terms of rigid fourfold, fivefold or sixfold coordination similar to mineral analogues. Instead, we observe a continuous statistical distribution of iron coordination numbers without any preference for particular local structural motifs.

## Data Availability

The raw neutron diffraction data are available at: https://doi.org/10.5291/ILL-DATA.6-03-441 [19]. Analysed data are available at the University of Bristol Research data repository, data.bris, at https://doi.org/10.5523/bris.w9d5ypz91ltv2wksargnm510s [46].
